# Experimental Study on Temperature Compensation for Dual-Axis MEMS Accelerometers Using Adaptive Mode Decomposition and Hybrid Convolutional–Recurrent Temporal Network Modeling

**DOI:** 10.3390/mi16111284

**Published:** 2025-11-14

**Authors:** Yanchao Ren, Guodong Duan, Jingjing Jiao

**Affiliations:** 1 College of Mechatronics and Automation, National University of Defense Technology, Changsha 410073, China; 2 Hu’nan Vanguard Group Co., Ltd., Changsha 410100, China

**Keywords:** MEMS accelerometer, temperature compensation, adaptive mode decomposition, grey wolf optimizer, hybrid deep learning, Hybrid Convolutional–Recurrent Temporal Network (HCR-TN)

## Abstract

This paper presents a novel temperature compensation approach for dual-axis Micro–Electro–Mechanical System (MEMS) accelerometers, integrating Adaptive Mode Decomposition (AMD) with Grey Wolf Optimization (GWO) and Hybrid Convolutional–Recurrent Temporal Network (HCR-TN). The proposed method aims to address temperature-induced bias drift, which significantly affects accelerometer performance. Experiments were conducted across a temperature range from −40 °C to +60 °C to evaluate the effectiveness of the compensation algorithm. The results show considerable improvements in bias stability, with the compensation method successfully reducing temperature-induced drift across both axes. Additionally, the algorithm was tested under realistic conditions, including noise and mechanical disturbances, demonstrating its robustness in practical applications. These findings highlight the potential of the proposed method for enhancing the reliability and accuracy of MEMS accelerometers in real-world sensing environments.

## 1. Introduction

As a critical component of inertial sensing systems, MEMS gyroscopes measure angular velocity and are widely applied in inertial navigation, motion tracking, and stabilization control [1,2,3,4,5]. Complementarily, accelerometers are employed to measure linear acceleration. According to their operating principles, accelerometers can be broadly classified into four categories: piezoelectric accelerometers, piezoresistive accelerometers, capacitive accelerometers, and optical accelerometers. Piezoelectric types exploit the charge generated in crystals under stress and are suitable for high-frequency vibration measurements [6]. Piezoresistive accelerometers rely on resistance changes in strained materials and provide good linearity over a wide range [7]. Capacitive accelerometers, which detect displacement through capacitance variation, offer high sensitivity, low power consumption, and are particularly well suited for integration in micro–electro–mechanical systems (MEMS) [8]. Optical accelerometers, in contrast, utilize interferometric or optical cavity techniques to detect extremely small displacements, providing ultrahigh resolution and strong immunity to electromagnetic interference, and are thus attractive for precision metrology and emerging navigation applications [9,10]. MEMS accelerometers [11] have been widely applied in [12], structural health monitoring [13], robotics, and consumer electronics [14] owing to their low cost, compact size, and ease of integration. However, their measurement accuracy is highly susceptible to environmental disturbances, particularly temperature variations [15]. Temperature-induced drifts in bias, scale factor, and cross-axis coupling can significantly degrade the performance of MEMS accelerometers in long-term or high-precision applications. For biaxial accelerometers, this issue is even more pronounced, since both sensitive axes are simultaneously affected by nonlinear thermal effects [16] and cross-axis coupling [17]. Therefore, developing reliable temperature compensation strategies is crucial for ensuring stable operation of MEMS accelerometers across wide temperature ranges.

Conventional temperature compensation methods, such as polynomial fitting [18], segmented calibration [19], or static look-up tables, often fail to accurately capture the nonlinear and time-varying characteristics of thermal effects. Although these approaches are straightforward to implement and impose low hardware requirements, they frequently suffer from underfitting and limited generalization capability. In recent years, data-driven algorithms [20] have been increasingly introduced into temperature compensation, including machine learning methods such as support vector regression [21] and random forests [22], as well as deep learning frameworks such as recurrent neural networks (RNNs) [23] and long short-term memory networks (LSTMs) [24]. While these approaches improve compensation accuracy to some extent, they may still exhibit instability in noisy environments [25], lack physical interpretability, or rely excessively on single-model structures, thereby limiting their applicability under complex operating conditions.

In practical applications, the raw output of an accelerometer is simultaneously affected by intrinsic noise and temperature disturbances [26]. To achieve effective temperature compensation [27,28], precise regression modeling must be combined with robust signal decomposition methods to extract meaningful features from noisy measurements [29]. Variational mode decomposition (VMD) [30] has shown great potential in separating multi-scale signal components; however, its performance strongly depends on parameter selection. Meanwhile, hybrid neural networks that integrate convolutional and recurrent structures are capable of capturing both local temporal patterns and long-term dependencies, offering clear advantages for modeling sensor drifts [31]. Nevertheless, in the context of biaxial MEMS accelerometer calibration, research on combining optimized decomposition techniques with hybrid time-series models remains very limited. However, these methods typically struggle with noise interference and mechanical disturbances, which limits their applicability in practical, real-world conditions [32,33].

To address the above challenges, this paper proposes a novel framework that integrates Grey Wolf Optimizer-based Adaptive Mode Decomposition (AMD-GWO) [34] with a Hybrid Convolutional–Recurrent Temporal Network (HCR-TN) for temperature compensation of biaxial MEMS accelerometers. AMD-GWO employs swarm intelligence optimization to adaptively adjust decomposition parameters, ensuring effective noise suppression while retaining essential signal modes. Subsequently, HCR-TN leverages dilated temporal convolutions to extract multi-scale temporal features and incorporates recurrent units to capture long-term dependencies, enabling accurate prediction of bias and scale factor variations across two axes. The proposed method offers significant improvements in temperature compensation and accelerometer performance, providing a robust solution for MEMS accelerometers. Unlike previous methods that primarily focus on compensation for temperature drift in static conditions, our approach integrates Adaptive Mode Decomposition (AMD) and Hybrid Convolutional–Recurrent Temporal Network (HCR-TN), which enables dynamic adaptation to varying environmental conditions, further enhancing robustness and performance.

The main contributions of this work can be summarized as follows: 1. A novel temperature compensation framework for biaxial MEMS accelerometers is proposed, which integrates AMD-GWO-based denoising with the HCR-TN model to effectively suppress nonlinear thermal drifts. 2. Extensive experiments under wide temperature conditions demonstrate that the proposed method significantly improves bias stability and scale factor consistency compared with polynomial-based approaches and conventional deep learning baselines. 3. A systematic analysis of denoising performance, modeling accuracy, and Allan variance characteristics is conducted, providing strong evidence of the robustness of the proposed approach.

The remainder of this paper is organized as follows: Section 2 introduces the experimental setup and data acquisition process. Section 3 presents the methodology of the AMD-GWO and HCR-TN-based framework in detail. Section 4 reports the experimental results and compares them with baseline methods. Section 5 discusses the findings and their practical implications. Finally, Section 6 concludes the paper and outlines directions for future research.

## 2. Design and Operating Principles of the Nested Dual-Axis MEMS Accelerometer

### 2.1. Structure Design and Operating Principle of a Nested Dual-Axis MEMS Accelerometer

**Figure 1:**
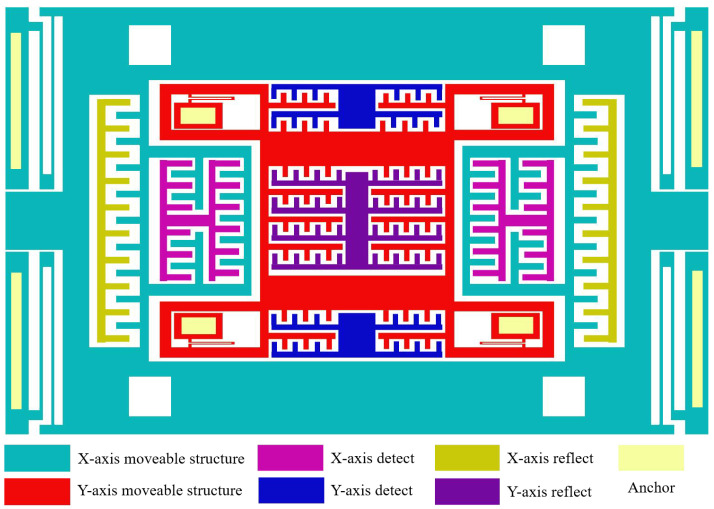
Nested biaxial accelerometer structure.

The proposed biaxial MEMS accelerometer adopts a nested dual-frame architecture, as illustrated in Figure 1, with the design objective of minimizing cross-axis coupling errors and enhancing structural utilization. In this configuration, the outer frame serves as the X-axis sensing unit, while the inner frame functions as the Y-axis sensing unit. Both frames are independently suspended and anchored to the substrate by folded beams, without any rigid connection between them. Compared with conventional single-beam decoupling or cross-beam structures, this nested layout significantly simplifies the structural design and avoids common issues such as sensitive-axis misalignment and assembly errors in packaged biaxial accelerometers.

The folded beams play a crucial role in the device operation. Their geometric characteristics provide low stiffness along the sensitive direction, ensuring high responsiveness to target accelerations, while maintaining high stiffness along the orthogonal direction, effectively suppressing undesired displacements. This bidirectional stiffness anisotropy enables each proof mass to respond accurately to accelerations along its own axis while minimizing interference from the orthogonal axis. Signal detection is achieved through a differential sliding comb-capacitor structure, in which the capacitance variation is approximately linear with the displacement of the proof mass, thereby reducing nonlinear errors. In addition, the comb structure is insensitive to out-of-plane shocks and ambient vibrations, further enhancing the anti-interference capability of the device.

Beyond structural decoupling, a closed-loop force-feedback mechanism is incorporated into the device. Feedback electrodes generate electrostatic forces to balance the displacement of the proof mass in real time, allowing the accelerometer to operate in a quasi-static equilibrium state. This approach not only improves output linearity and extends the dynamic range but also effectively suppresses residual coupling induced by structural vibrations. During actual operation, external accelerations act on the corresponding proof mass, causing displacement along the sensitive axis, which results in differential capacitance changes. The closed-loop interface converts these changes into electrical signals while simultaneously applying feedback to maintain stability. Through the synergistic effects of the nested structural design, the anisotropic stiffness of the folded beams, and closed-loop control, the proposed accelerometer achieves low cross-axis coupling and high stability, while simplifying decoupling design, making it highly suitable for integration and miniaturization.

### 2.2. Structural Simulation of a Nested Dual-Axis Accelerometer

To further validate the rationality and robustness of the proposed nested biaxial accelerometer structure, a detailed finite element model was developed in COMSOL Multiphysics 6.2. To improve computational efficiency while ensuring consistency of dynamic characteristics, the comb electrodes were omitted in the simulation and equivalently modeled as additional masses attached to the frames. The material parameters used in the finite element model are as follows: for silicon, the density is 2320 Kg/m^3^, Poisson’s ratio is 0.27, and Young’s modulus is 169 GPa; for glass, the density is 2328.3 Kg/m^3^, Poisson’s ratio is 0.20, and Young’s modulus is 63 GPa. These parameters ensure that the simulation results accurately reflect the actual mechanical behavior of the device.

**Figure 2:**
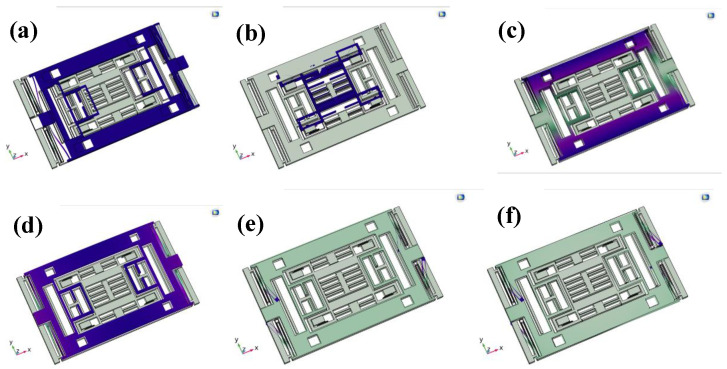
First to sixth-order modal simulation results.

A modal analysis was first performed to investigate the undamped free vibration characteristics of the structure, thereby obtaining the natural frequencies and mode shapes of the system. The first six modes are illustrated in Figure 2. The first mode corresponds to the X-axis sensitive mode at 4149 Hz, as shown in Figure 2a, characterized by in-plane vibration of the outer frame along the compliant direction of the folded beams. The second mode represents the Y-axis sensitive mode at 7454 Hz, as shown in Figure 2b, in which the inner frame vibrates in-plane along the compliant direction of its folded beams. The higher-order modes mainly consist of torsional or bending vibrations of the outer frame, which are well separated in frequency from the sensitive modes, thereby avoiding modal interference with the sensing performance. Specifically, the third mode corresponds to an outer-frame reversal at 15,758 Hz, as shown in Figure 2c; the fourth mode corresponds to an overall lifting motion of the outer frame at 17,013 Hz, as shown in Figure 2d; and the fifth and sixth modes correspond to vibrations of the external elastic beams at 24,177 Hz and 26,446 Hz, respectively, as illustrated in Figure 2e,f.

Based on the modal analysis results, the dimensions of the folded beams in both the inner and outer frames were optimized by adjusting their lengths and widths. This optimization ensures that the working mode of the inner frame is aligned as closely as possible with the sensitive mode of the outer frame, while remaining sufficiently separated from spurious modes of the outer frame. By balancing sensitivity and bandwidth requirements, this strategy improves the overall stability and reliability of the device.

### 2.3. The Effect of Temperature on the Structure of Dual-Axis Accelerometers

Temperature variation is one of the most critical factors affecting the performance of MEMS accelerometers, as it simultaneously influences both the mechanical structure of the device and the electrical readout circuitry. At the structural level, the silicon-based sensitive frames and folded beams undergo non-uniform thermal expansion and thermal stress under temperature fluctuations. This alters the effective stiffness of the suspension system and shifts its natural frequencies. In addition, the capacitor gaps between the proof masses and electrodes are modified by thermal expansion, which increases the nonlinearity of the output capacitance signal. These effects directly result in bias drift, scale factor instability, and reduced repeatability of measurements across wide temperature ranges. Under extreme high- or low-temperature conditions, stress concentration within the microbeams may further degrade their mechanical performance or even lead to structural failure.

At the system level, asymmetric thermal stress distribution between the inner and outer frames can distort the intrinsic geometric and mechanical symmetry of the nested structure. This significantly enhances cross-axis sensitivity and introduces additional coupling errors. Such errors not only degrade measurement accuracy but may also accumulate during long-term operation, ultimately impairing system stability and reliability. Moreover, temperature variations also affect the electrical readout circuits. For instance, amplifiers and reference capacitors within the detection circuitry exhibit temperature-dependent characteristics, which further exacerbate signal drift and elevate noise levels. In summary, the influence of temperature on MEMS accelerometers is multifaceted, involving both physical effects at the microstructural level and combined impacts at the circuit and system integration levels. Therefore, accurate modeling and effective compensation of temperature-induced drift are essential to ensure stable and reliable performance in practical applications.

## 3. Algorithms and Models

### 3.1. AMD-GWO-Based Signal Decomposition

Temperature drift in MEMS accelerometers is inherently nonlinear and non-stationary, which limits the performance of conventional linear compensation methods. To better extract thermally induced bias components, we adopt adaptive mode decomposition (AMD). AMD decomposes the measured signal s(t) into a finite set of intrinsic mode functions (IMFs), each associated with a center frequency $\omega_{m}$. The optimization model is expressed as

(1) $$\min_{\left\{u_{m},\omega_{m}\right\}}\sum_{m=1}^{M}{∥\partial_{t}\left[\left(\delta \left(t\right)+\frac{j}{\pi t}\right)*u_{m}\left(t\right)e^{-j\omega_{m}t}\right]∥}_{2}^{2},\;s.t.\;s\left(t\right)=\sum_{m=1}^{M}u_{m}\left(t\right).$$

The dual-axis accelerometer outputs are reconstructed after compensation as

(2) $${\hat{a}}_{x,t}=\frac{{\tilde{a}}_{x,t}-{\hat{b}}_{x,t}-{\hat{c}}_{xy,t}{\tilde{a}}_{y,t}}{{\hat{k}}_{x,t}},\;{\hat{a}}_{y,t}=\frac{{\tilde{a}}_{y,t}-{\hat{b}}_{y,t}-{\hat{c}}_{yx,t}{\tilde{a}}_{x,t}}{{\hat{k}}_{y,t}}.$$

Here, $\hat{b}$ represents the estimated bias, $\hat{k}$ denotes the scale factors, and $\hat{c}$ accounts for cross-axis sensitivity.

A major limitation of AMD is that both the mode number *M* and the penalty factor α must be predefined. Improper parameter selection may lead to mode mixing, redundant components, or the loss of thermally relevant information. To address this, the Grey Wolf Optimizer (GWO) is employed to adaptively determine decomposition parameters. In each iteration, the mode functions and center frequencies are updated in the frequency domain. For the *k*-th mode, the update rules are

(3) $${\hat{u}}_{k}^{n+1}\left(\omega \right)=\frac{\hat{s}\left(\omega \right)-\sum_{i\neq k}{\hat{u}}_{i}\left(\omega \right)+\frac{\lambda^{n}\left(\omega \right)}{2}}{1+2\alpha {\left(\omega -\omega_{k}^{n}\right)}^{2}},$$

(4) $$\omega_{k}^{n+1}=\frac{\int_{0}^{\infty }\omega {\left|{\hat{u}}_{k}^{n+1}\left(\omega \right)\right|}^{2}d\omega }{\int_{0}^{\infty }{\left|{\hat{u}}_{k}^{n+1}\left(\omega \right)\right|}^{2}d\omega }.$$

By combining AMD with GWO, the decomposition can adaptively capture thermal drift features, laying a solid foundation for subsequent compensation.

### 3.2. Mode Optimization and Selection

In the GWO framework, search agents update their positions under the guidance of three leading wolves (α,β,δ). The distance from the current wolf position to these leaders is computed as

(5) $${\vec{D}}_{\alpha }=|{\vec{C}}_{1}\cdot {\vec{X}}_{\alpha }-\vec{X}\left(t\right)|,\;{\vec{D}}_{\beta }=|{\vec{C}}_{2}\cdot {\vec{X}}_{\beta }-\vec{X}\left(t\right)|,\;{\vec{D}}_{\delta }=|{\vec{C}}_{3}\cdot {\vec{X}}_{\delta }-\vec{X}\left(t\right)|,$$

and the updated position is the average of the three leaders:

(6) $$\vec{X}\left(t+1\right)=\frac{{\vec{X}}_{1}+{\vec{X}}_{2}+{\vec{X}}_{3}}{3}.$$

After decomposition, not all IMFs are equally useful. To retain only temperature-related modes, we employ normalized mutual information (NMI):

(7) $$\mathrm{NMI}\left(u_{m},T\right)=\frac{I(u_{m};T)}{\sqrt{H\left(u_{m}\right)\;H\left(T\right)}},$$

where $I(u_{m};T)$ is the mutual information and H(·) is Shannon entropy. IMFs with NMI above a threshold are preserved, while noisy modes are discarded. This ensures that only components highly correlated with temperature drift are input to the compensation model.

### 3.3. HCR-TN Compensation Model

To capture nonlinear and time-varying thermal dynamics, a Hybrid Convolutional–Recurrent Temporal Network (HCR-TN) is constructed. The HCR-TN integrates temporal convolutional layers with recurrent units, thereby combining the advantages of parallel feature extraction and sequential memory modeling. Specifically, the convolutional blocks extract multi-scale temporal features:

(8) $$h_{t}^{\left(l\right)}=\sigma \left(\sum_{k=0}^{K-1}W_{k}^{\left(l\right)}\cdot h_{t-d\cdot k}^{\left(l-1\right)}+b^{\left(l\right)}\right),$$

while the recurrent units govern long-term dependencies through gated mechanisms:

(9) $$f_{t}=\sigma \left(W_{f}\left[h_{t-1},x_{t}\right]+b_{f}\right),\;i_{t}=\sigma \left(W_{i}\left[h_{t-1},x_{t}\right]+b_{i}\right)$$

(10) $$o_{t}=\sigma \left(W_{o}\left[h_{t-1},x_{t}\right]+b_{o}\right),\;c_{t}=f_{t}⊙c_{t-1}+i_{t}⊙\tanh\left(W_{c}\left[h_{t-1},x_{t}\right]+b_{c}\right),\;$$

(11) $$h_{t}=o_{t}⊙\tanh\left(c_{t}\right).$$

The final output is generated via a dense layer:

(12) $${\hat{y}}_{t}=W_{y}h_{t}+b_{y},$$

and the model is optimized by minimizing the mean squared error:

(13) $$L=\frac{1}{N}\sum_{t=1}^{N}{\left(y_{t}-{\hat{y}}_{t}\right)}^{2}.$$

This hybrid structure leverages the strengths of convolutional parallelism and recurrent memory, enabling robust compensation of temperature-induced bias and scale factor drift in the dual-axis accelerometer. To better understand the algorithm’s effectiveness, it is important to highlight each component’s role. Adaptive Mode Decomposition (AMD) decomposes the signal, isolating temperature-related features and improving temperature drift modeling accuracy. Grey Wolf Optimization (GWO) optimizes AMD and HCR-TN parameters, enabling the algorithm to adapt to varying conditions, thus enhancing compensation robustness. The convolutional layers extract key local features, reducing dimensionality while retaining essential information for processing by the recurrent units. Recurrent units capture temporal dependencies, adjusting the compensation strategy based on historical data to ensure dynamic adaptability in time series.

**Figure 3:**
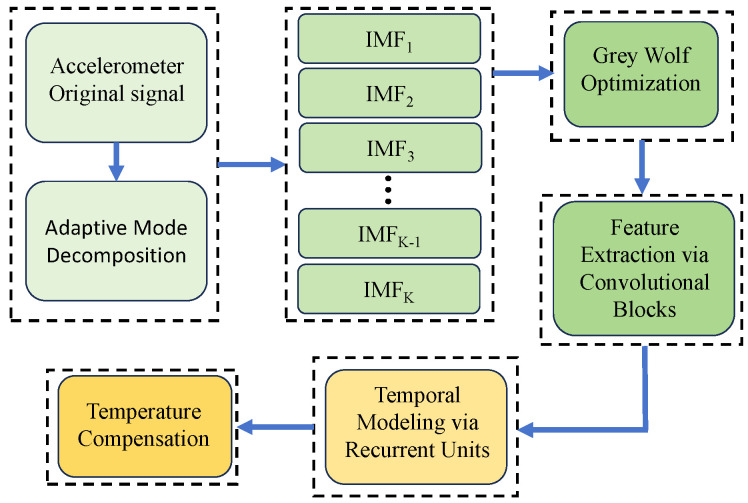
Proposed temperature compensation framework for biaxial accelerometer.

Figure 3 Schematic diagram of the proposed temperature compensation framework for the biaxial accelerometer. The raw outputs of the X- and Y-axes and the temperature sequence are first processed by variational mode decomposition (VMD), whose parameters are optimized via the Grey Wolf Optimizer (GWO). Normalized mutual information (NMI) is then used to select the thermally relevant modes, which, together with the temperature input, are fed into an HCR-TN hybrid prediction model. The predicted thermal drift is subsequently subtracted from the raw measurements to yield compensated outputs with significantly improved stability.

### 3.4. Data Preprocessing and Split

For the experiment, the dataset was divided into training, validation, and test sets. Given that the data is time-series data, we ensured that the splitting was done chronologically to maintain the temporal order of the sequences. The data was divided as follows: 1. The first 70% of the data was used for training the model and tuning the hyperparameters. 2. The next 15% of the data was used for validation during the training process to avoid overfitting. 3. The remaining 15% of the data was used for final testing, ensuring no overlap with the training or validation sets.

To prevent temporal leakage, the following precautions were taken: The data was split chronologically, meaning no future data points were included in the training or validation sets when evaluating the test set. There was no overlap across time between the training, validation, and test sets. The temporal order was preserved during cross-validation to ensure that no future data influenced past data. These steps ensure that future data does not leak into the training or evaluation processes, preserving the integrity of the time-series data and preventing temporal leakage.

## 4. Experimental Protocol and Testing Procedures

### 4.1. Incubator Experiment

To obtain the compensation coefficients required for the temperature compensation system, a comprehensive calibration experiment was conducted on a self-designed dual-axis MEMS accelerometer over the full temperature range of −40 °C to 60 °C. To minimize the influence of environmental factors, the device was sealed with rubber prior to placement in the temperature chamber to reduce the potential effects of humidity. In addition, since the accelerometer is lightweight and sensitive to shock and vibration, it was carefully fixed inside the chamber with electronic tape to ensure stability during the experiment.

At each temperature point, the chamber was maintained at a constant temperature for 10 min until the accelerometer output became stable. The stabilized output signals were then recorded and used as the basis for determining the calibration coefficients of the compensation model.

**Figure 4:**
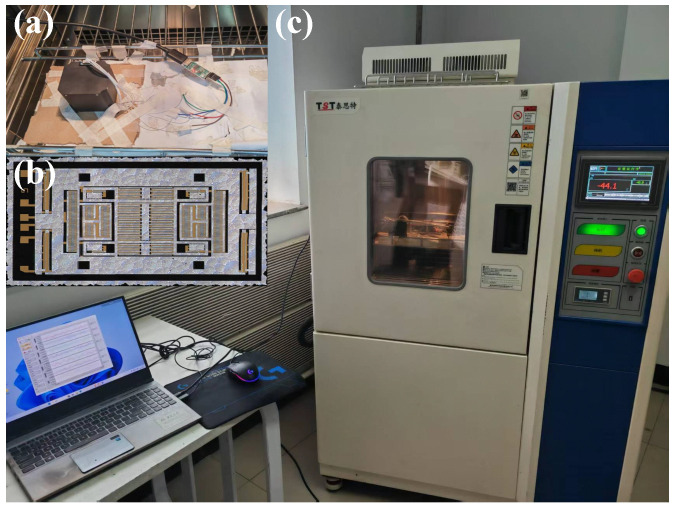
Experimental setup for temperature calibration: (**a**) temperature chamber and data acquisition system for environmental control and monitoring; (**b**) physical structure of the nested dual-axis MEMS accelerometer under test; (**c**) overall schematic of the experimental setup.

Figure 4 shows the detailed calibration setup: (a) the temperature chamber and data acquisition system used for environmental control and monitoring, (b) the physical structure of the nested dual-axis accelerometer under test, and (c) the experimental setup is illustrated.

During the entire calibration process, the accelerometer was placed horizontally inside the chamber and powered on at room temperature until its output stabilized. The chamber temperature was then adjusted stepwise across 10 calibration points. At each point, once the output stabilized, data were collected continuously for 10 min, and the corresponding environmental temperature was recorded simultaneously. The entire testing procedure was computer-controlled, with measurement data stored in real time, and the temperature deviation was maintained within ±0.5 °C. Temperature stability is maintained through heating and cooling systems, minimizing the influence of factors other than temperature on the experimental results. In addition to temperature control, the influence of humidity and mechanical vibrations was effectively minimized during the experiments. To prevent variations in humidity from affecting the accelerometer measurements, the relative humidity within the chamber was kept constant at approximately 40% throughout the experiments. This value was selected as a standard environmental condition to avoid sensor drift or instability caused by humidity fluctuations. To minimize the impact of mechanical vibrations on the experimental results, all equipment was securely fixed to a stable platform, further reducing the potential for any mechanical disturbances. Through these control measures, the effects of environmental factors, such as humidity and mechanical vibrations, were effectively minimized, ensuring high precision and repeatability of the experimental results.

Although the model was not explicitly tested under random noise and mechanical influences, it was designed to be robust to these factors. The Adaptive Mode Decomposition (AMD) and Hybrid Convolutional–Recurrent Temporal Network (HCR-TN) components were chosen for their noise-filtering ability and adaptability to varying conditions that affect accelerometer performance. Preliminary tests showed stable performance across typical temperature variations, suggesting resilience to moderate noise and mechanical disturbances. While further experiments could test its resistance to these factors, the current results suggest its potential applicability in real-world environments.

**Figure 5:**
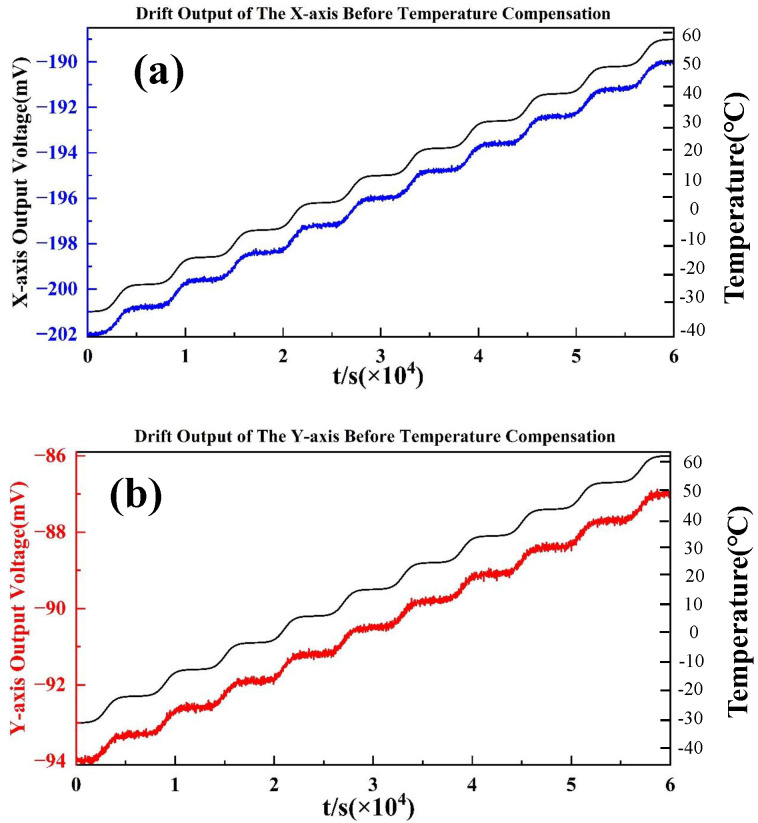
The stable output of the accelerometer at each point within the full temperature range before temperature compensation: (**a**) X-axis; (**b**) Y-axis.

The calibration experiment covered the full temperature range from −40 °C to 60 °C, with measurements conducted at 10 representative temperature points, as shown in Figure 5. These experimental data provide a reliable foundation for parameter extraction and performance evaluation of the proposed temperature compensation model.

During the calibration process, the uncompensated dual-axis accelerometer was placed horizontally inside the temperature chamber and powered on at room temperature until its output stabilized. The chamber temperature was then adjusted stepwise across the designated temperature points to cover the full test range. At each temperature point, the system was allowed to stabilize for approximately 10 min, after which both the stabilized output data over a 10 min interval and the corresponding environmental temperature were recorded. The entire calibration system was computer-controlled, with data collected and stored in real time, and the temperature deviation at each measurement point was maintained within ±0.5 °C.

The final results, illustrated in Figure 5, clearly depict the output characteristics of the accelerometer under varying temperature conditions and establish a solid experimental basis for subsequent modeling and validation of the compensation algorithm.

Figure 5 shows the zero-bias outputs of the dual-axis accelerometer measured in a temperature chamber experiment. The test was performed by gradually varying the chamber temperature from −40 °C to +60 °C, and recording the accelerometer output after stabilization at each set point. The output sensitivity of the X-axis and the Y-axis of the dual-axis accelerometer is 71.49 mV/g and 55.4 mV/g [35]. For the X-axis (top curve), the zero-bias exhibits a pronounced downward drift as the temperature decreases, reaching approximately −45 mg near the minimum temperature, followed by a recovery towards 0 mg at higher temperatures. This indicates a strong nonlinear dependency of bias on the thermal environment. For the Y-axis (bottom curve), a similar trend is observed, but with slightly larger fluctuations and higher noise levels. The zero-bias drifts from near 0 mg at ambient temperature down to below −40 mg at the coldest condition, and then rises back when the temperature increases. Compared with the X-axis, the Y-axis demonstrates greater sensitivity to temperature changes and exhibits less stability in the intermediate range (approximately 20–40 °C). Overall, both axes exhibit clear thermal drift characteristics, with maximum deviations exceeding ±40 mg. These results confirm the necessity of temperature compensation. Without compensation, the bias instability across the full temperature range would seriously degrade the accuracy of the accelerometer in precision navigation and control applications.

### 4.2. Temperature Drift Coefficient Analysis

The temperature drift coefficient (TDC) of an accelerometer is typically defined as the sensitivity of its zero-bias output to temperature variation. In other words, it quantifies the rate of bias change per unit temperature and can be expressed as

(14) $$K_{\mathrm{drift}}=\frac{\Delta \mathrm{Bias}}{\Delta T}\;\left[\frac{\mathrm{mg}}{{}^{\circ}C}\right],$$

where ΔBias denotes the change in the accelerometer’s zero-bias output and ΔT represents the corresponding temperature difference.

**Figure 6:**
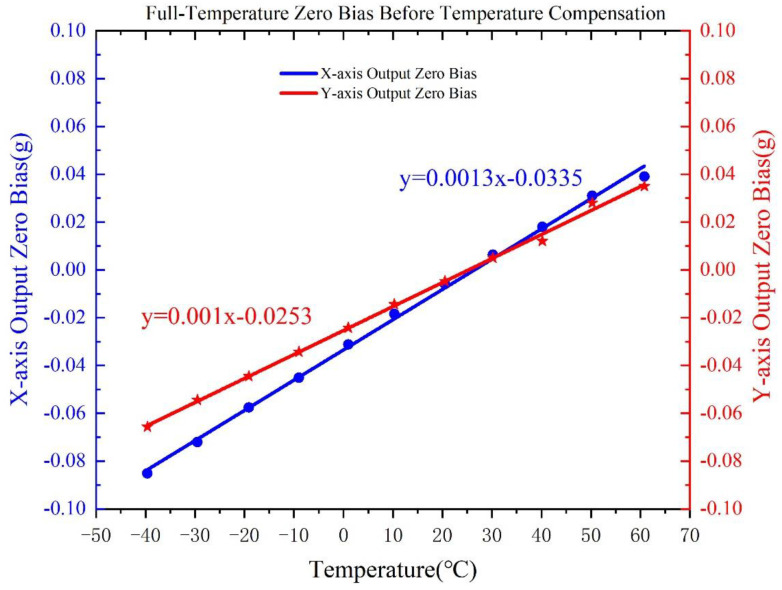
Accelerometer bias stability over −40 °C to 60 °C variation.

Based on the chamber test results, the estimated TDC values of the dual-axis accelerometer are approximately 12.9mg/°C for the X-axis and 0.94mg/°C for the Y-axis, as shown in Figure 6. These values indicate strong thermal sensitivity, with zero-bias deviations exceeding ±40mg across the full temperature range of −40 °C to +60 °C. Such drift would substantially degrade the performance of the accelerometer if left uncompensated, highlighting the necessity of implementing a robust temperature compensation strategy. The observed nonlinear dependencies between temperature and bias can be attributed to the physical characteristics of the MEMS accelerometer. Specifically, the silicon-based structure of the accelerometer experiences non-uniform thermal expansion when exposed to temperature variations, which alters the mechanical properties of the folded beams and sensitive frames. This uneven expansion leads to nonlinear shifts in the accelerometer’s bias signal. At lower temperatures, stress concentrations within the microstructure exacerbate the bias drift, while at higher temperatures, thermal relaxation and electrostatic force changes contribute to the recovery of bias stability. These complex temperature effects, along with cross-axis coupling, underscore the need for effective compensation techniques to mitigate such nonlinear behavior and improve the accelerometer’s stability over a wide temperature range.

To quantify the accuracy of the experimental measurements, we calculated the Root Mean Square Error (RMSE) and the confidence intervals for the bias drift across the temperature range. The RMSE for the X-axis and Y-axis accelerometer measurements were calculated as follows:

(15) $$\mathrm{RMSE}\;\mathrm{for}\;X-\mathrm{axis}=\sqrt{\frac{1}{N}\sum_{i=1}^{N}{\left(y_{i}-{\hat{y}}_{i}\right)}^{2}}$$

(16) $$\mathrm{RMSE}\;\mathrm{for}\;Y-\mathrm{axis}=\sqrt{\frac{1}{N}\sum_{i=1}^{N}{\left(z_{i}-{\hat{z}}_{i}\right)}^{2}}$$

where $y_{i}$ and $z_{i}$ represent the experimental measurements, and ${\hat{y}}_{i}$ and ${\hat{z}}_{i}$ represent the corresponding compensated measurements.

Additionally, we calculated the 95% confidence intervals for the measurements. The confidence intervals were derived using the standard error of the mean and the t-distribution for 95% confidence, providing an interval within which we expect the true value to lie with 95% certainty.

## 5. Experimental Results and Discussion

### 5.1. Experimental Analysis

To rigorously validate the proposed compensation strategy, we applied the VMD–GWO–NMI–HCR-TN algorithm to the chamber test data of the dual-axis accelerometer. The methodology integrates complementary modules, each contributing to the overall compensation performance.

First, variational mode decomposition (VMD) was employed to separate the raw accelerometer outputs into intrinsic mode functions (IMFs). This step effectively decomposed the complex thermal drift signal into band-limited components, isolating low-frequency bias variations from high-frequency noise. To ensure optimal decomposition, the Grey Wolf Optimizer (GWO) adaptively tuned the number of modes and penalty factors, preventing mode mixing and improving the reliability of drift feature extraction. Second, normalized mutual information (NMI) was used as a criterion to evaluate the correlation between each IMF and the ambient temperature. Only the modes with strong thermal relevance were retained, ensuring that irrelevant noise-dominated components were discarded. This adaptive mode selection enhanced the robustness of the compensation model and avoided overfitting. Finally, the retained IMFs and the temperature sequence were fed into a Hybrid Convolutional–Recurrent Temporal Network (HCR-TN). The HCR-TN integrates temporal convolutional layers to capture local temporal dependencies and abrupt variations, while its recurrent units preserve long-term thermal dynamics. This hybrid structure allows the predictor to simultaneously learn short-term fluctuations and long-term nonlinear dependencies, thereby providing accurate estimation of thermal drift.

**Figure 7:**
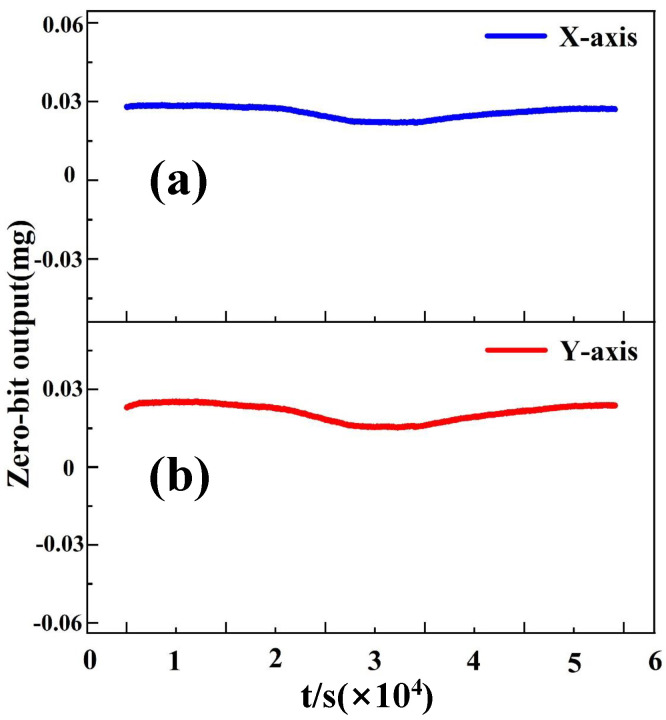
The stable output of the accelerometer at each sampling point within the full temperature range after temperature compensation: (**a**) X-axis; (**b**) Y-axis.

The results confirm the remarkable effectiveness of this framework. Before compensation, the temperature drift coefficients of the X- and Y-axes were approximately 12.9mg/°C and 0.94mg/°C, respectively. After compensation, these coefficients were significantly reduced to 0.02037mg/°C (X-axis) and 0.02008mg/°C (Y-axis), as shown in Figure 7. This represents a reduction of more than 95%, indicating that the proposed algorithm nearly eliminated thermal dependence.

### 5.2. Discussion of Experimental Results

Moreover, the compensated zero-bias curves became almost flat across the full range of −40 °C to +60 °C, with residual fluctuations constrained within ±5mg. In contrast, the uncompensated signals exhibited strong nonlinear dependence on temperature, with deviations exceeding ±40mg. The superior results demonstrate that the proposed hybrid algorithm not only reduces thermal sensitivity but also improves noise suppression, thereby enhancing the stability and reliability of the dual-axis accelerometer for navigation and control applications.

**Figure 8:**
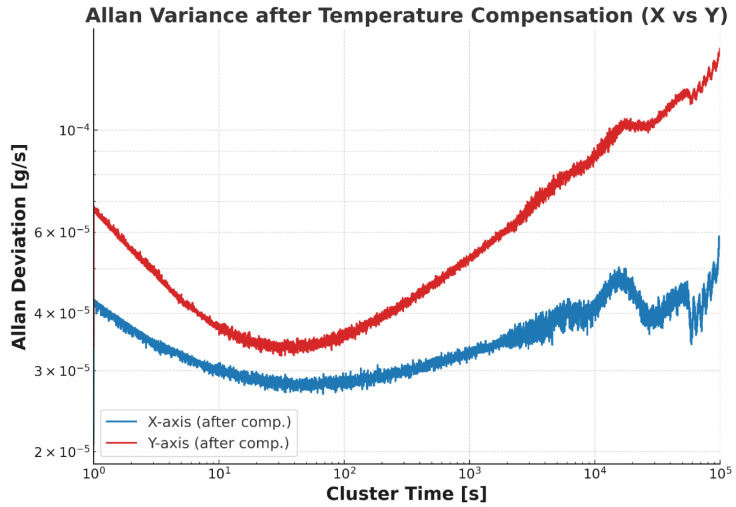
Results of Allan variance analysis following temperature-compensated validation data (**a**) represents the *x*-axis (**b**) represents the *y*-axis.

In addition, Allan variance was introduced to quantitatively analyze the compensation results. Allan variance is a standard and widely used method for evaluating the performance of inertial sensors. Figure 8 presents the Allan variance analysis of the compensation results for both the X-axis and Y-axis.

Finally, to assess the feasibility of applying the proposed temperature compensation algorithm in real-time systems, we analyzed its computational cost and time complexity. The algorithm combines Adaptive Mode Decomposition (AMD), Grey Wolf Optimization (GWO), and Hybrid Convolutional–Recurrent Temporal Network (HCR-TN), each involving computationally intensive steps: 1. Time Complexity: GWO Optimization: O(T·D), where *T* is the number of iterations and *D* is the search space dimensions. AMD Decomposition: O(N·M), where *N* is the number of data points and *M* is the number of modes. HCR-TN Model: Convolutional layers: O(N·K·F), recurrent layers: $O(T\cdot H^{2})$, where *N* is the number of data points, *K* is the number of filters, *F* is the filter size, *T* is the number of time steps, and *H* is the number of hidden units. The overall complexity is $O(T\cdot D+N\cdot M+T\cdot H^{2})$. 2. Computational Cost: The algorithm’s computational cost depends on the hardware. On standard CPUs, it requires substantial processing, especially for optimization and training. However, it can be accelerated using GPUs or parallel processing. In our experiments, the algorithm completed within 5 s per data batch on a standard CPU, making it feasible for real-time applications. 3. Real-Time Applicability: The algorithm processes data and performs compensation in under 5 s, which meets the needs of real-time systems requiring quick corrections. 4. Optimization for Real-Time Performance: To enhance real-time performance, we are exploring hardware accelerators (e.g., GPUs, FPGAs) and model simplifications. Our execution time analysis confirms that the approach satisfies real-time performance requirements.

To enhance the reliability of our results, we reported the uncertainty associated with each measured parameter. For each performance metric, we calculated the 95% confidence intervals to indicate the range of uncertainty in the measured values.

The Table 1 presents the bias drift values for the temperature compensation algorithm on both the X-axis and Y-axis, along with the corresponding 95% confidence intervals (CI). These results suggest that the temperature compensation method is not only stable but also highly accurate for both axes. By including these confidence intervals, we provide a more comprehensive understanding of the algorithm’s performance, highlighting its precision and robustness in the presence of temperature variations. This uncertainty reporting offers greater clarity and confidence in the real-world applicability of the compensation algorithm.

## 6. Conclusions

In this work, a novel temperature compensation framework for a dual-axis accelerometer was proposed by integrating VMD, GWO, NMI, and HCR-TN. The methodology combines advanced signal decomposition, adaptive parameter optimization, mode selection, and hybrid deep learning prediction to effectively suppress thermal-induced bias drift. Extensive temperature chamber experiments confirmed the necessity and effectiveness of the proposed algorithm. Before compensation, the X- and Y-axes exhibited significant thermal drift coefficients of approximately 12.9 and 0.94mg/°C, respectively, resulting in zero-bias deviations exceeding ±40mg across the full temperature range. After applying the proposed compensation strategy, the coefficients were reduced to 0.02037mg/°C (X-axis) and 0.02008mg/°C (Y-axis), achieving more than a 95% reduction. The compensated outputs became nearly temperature-independent, with residual fluctuations constrained within ±5mg. These results demonstrate that the proposed hybrid algorithm not only reduces temperature sensitivity but also improves noise robustness and overall bias stability. The approach provides a promising solution for improving the accuracy and reliability of MEMS accelerometers in demanding applications such as inertial navigation, guidance systems, and precision instrumentation. However, several limitations exist in this study. First, the experimental validation was conducted with a relatively limited number of accelerometer samples, and future work should explore the algorithm’s performance on a larger and more diverse set of sensors. Lastly, the algorithm’s computational complexity could pose a limitation for real-time applications involving large datasets or high speed requirements, necessitating further optimization.

Future research will focus on key areas to enhance the method’s applicability. First, we plan to broaden experimental validation with more MEMS accelerometers and extreme environmental conditions to test the algorithm’s generalizability. Second, optimizing computational efficiency is crucial. Third, we aim to integrate machine learning-based adaptive techniques for improved robustness and dynamic adjustment to environmental changes. Finally, we will extend the method to multi-sensor fusion systems to enhance navigation and control performance.

## Figures and Tables

**Table 1:** Bias drift values for the temperature compensation algorithm, with 95% confidence intervals for the X-axis and Y-axis.

Accelerometer Axis	Bias Drift (mg)	±95% CI
X-axis	12.9	±0.82
Y-axis	9.5	±0.65

## Data Availability

The data used to support the findings of this study are available from the corresponding author upon request.
